# Proteoglycans as Biomarkers of Medial Degeneration in Acute Stanford Type A Aortic Dissection

**DOI:** 10.31083/RCM45189

**Published:** 2025-12-24

**Authors:** Ghufran Bagaber, Wenyu Song, Guangguo Fu, Kui Hu, Zhe Wang, Yubin Hu, Lai Wei, Jinmiao Chen

**Affiliations:** ^1^Department of Cardiovascular Surgery, Zhongshan Hospital, Fudan University, 200032 Shanghai, China; ^2^Shanghai Medical College, Fudan University, 200032 Shanghai, China; ^3^Department of Cardiovascular Surgery, Guizhou Provincial People’s Hospital, 550002 Guiyang, Guizhou, China

**Keywords:** acute Stanford type A aortic dissection, thoracic aortic dissection, serum biomarker, proteoglycans, aggrecan, versican, lumican

## Abstract

Acute Stanford type A aortic dissection (ATAAD) is a life-threatening cardiovascular emergency that demands prompt and accurate diagnosis due to a high associated mortality. Although imaging remains the diagnostic gold standard, the limited accessibility and time sensitivity of this technique underscore the need for reliable serum biomarkers. D-dimer is the most widely used biomarker, offering high sensitivity; however, the limited specificity of using D-dimer has prompted a search for novel biomarkers with greater diagnostic precision. Interestingly, proteoglycans (PGs) are essential constituents of the extracellular matrix (ECM) and have emerged as promising candidates for ATAAD, as PGs are released into the circulation during medial degradation, a defining histological feature of ATAAD. Moreover, emerging evidence suggests that specific PGs exhibit favorable specificity and stability, potentially enabling distinction between ATAAD and other acute cardiovascular syndromes. Additionally, in contrast to D-dimer, which is rapidly cleared within 8 hours, certain PGs, such as aggrecan, remain stable for up to 72 hours, offering an advantage for detecting ATAAD in patients presenting beyond the early acute phase. This review summarizes the potential of aortic PGs as biomarkers of medial degeneration and circulating PGs as serum diagnostic markers of ATAAD. Future research is warranted to establish PGs as clinically reliable biomarkers, with the potential to enhance current diagnostic frameworks and support an earlier, more accurate identification of ATAAD.

## 1. Introduction

Thoracic aortic dissection is a catastrophic cardiovascular emergency caused by intimal tearing and false lumen formation [1]. Briefly, it is classified into Stanford type A aortic dissection (ATAAD), which involves the ascending aorta, and otherwise Stanford type B. The antegrade and retrograde propagation of ATAAD may trigger multiple complications (i.e., acute aortic regurgitation, myocardial ischemia, cardiac tamponade, stroke, or organ malperfusion) and even aortic rupture and death [1, 2]. Open surgical repair remains the guideline-recommended therapy for ATAAD [1]. Despite advances in imaging and surgical procedures, untreated ATAAD still has a mortality rate of 40–50% within the first 48 hours [3].

Accurate and timely diagnosis is essential for early surgical interventions of ATAAD. The primary symptoms are often chest pains, which may also be considered as acute coronary syndrome (ACS) or pulmonary embolism (PE) [3, 4], thereby contributing to diagnostic delays or even misdiagnosis [5]. When ATAAD involves coronary arteries, the patients may display syndromes of ACS with ST-segment elevation on the electrocardiogram and abnormal myocardial enzymes. This may further enhance the challenge for distinguishing ATAAD and ACS. Notably, approximately 80% of misdiagnosed ATAAD cases are initially mistaken for ACS, followed by the inappropriate administration of thrombolytic therapy and thereby worsened outcomes [6]. Current diagnosis of ATAAD relies on imaging modalities such as computed tomography angiography (CTA), magnetic resonance angiography (MRA), and transesophageal echocardiography (TEE) [1]. Although these techniques offer high diagnostic accuracy, they are inherently constrained by several factors including limited accessibility, time-consuming patient-transferring [7], ionizing radiation exposure [8], and contrast-induced nephropathy risk [9]. Typical ATAAD is marked by a dilated aortic root, ascending aorta or aortic arch with an intimal flap. However, conventional computed tomography (CT) scans may miss highly suspected ATAAD patients with atypical CT images [10, 11]. These limitations emphasize the necessity for developing highly sensitive and specific biomarkers to enhance the early diagnosis of ATAAD.

Proteoglycans (PGs), essential ECM components comprising a core protein linked to glycosaminoglycan (GAG) chains, contribute to aortic structural integrity, cellular signaling and ECM remodeling [12, 13, 14]. In recent years, PGs have garnered attention as potential biomarkers for ATAAD. On one side, acute aortic tearing leads to the destruction of normal aortic walls and the release of PGs into the blood circulation. On the other hand, dissection formation triggers vascular remodeling marked by the accumulation of PGs. This review compares the potential of specific PGs as biomarkers for ATAAD. Future progress of biomarker research of PGs may provide novel insights into early diagnosis of ATAAD in the emergency.

## 2. Limitations of Existing Biomarkers in ATAAD

Despite their diagnostic value, currently available biomarkers for ATAAD present several limitations that restrict their standalone diagnostic utility. Intimal tearing is the defining anatomic feature of ATAAD, triggering thrombosis in the false lumen. D-dimer is a fibrin degradation product released during fibrinolysis and is a classical biomarker of thrombosis [15]. Several meta-analyses consistently report high pooled sensitivities for D-dimer in ATAAD, typically ranging from 95% to 98%, making it a valuable rule-out test in patients with low clinical suspicion [16, 17]. However, its clinical utility is constrained by poor specificity ranging from 42% to 70%, given its elevation in other thrombotic and inflammatory conditions, including ACS and PE [15, 18, 19], deep vein thrombosis [20, 21, 22] and disseminated intravascular coagulation [15].

In emergency departments, where patients often present with nonspecific symptoms, D-dimer’s specificity is frequently compromised due to the high prevalence of alternative conditions that elevate D-dimer, such as PE, deep vein thrombosis (DVT), ACS, and systemic inflammation [18, 19, 20, 21, 22, 23]. In contrast, in specialized cardiovascular centers where pre-test probability is higher, the positive predictive value of D-dimer improves, though specificity remains moderate.

Patient-specific factors further affect D-dimer’s diagnostic performance. In elderly patients, age-related increases in baseline D-dimer levels reduce specificity, necessitating the use of age-adjusted cutoffs (e.g., age × 0.01 µg/mL) to maintain diagnostic accuracy [24, 25]. Conversely, in younger patients, particularly those with connective tissue disorders (e.g., Marfan syndrome, Ehlers-Danlos syndrome), D-dimer levels may be deceptively low or normal despite severe dissection, owing to atypical presentations or delayed fibrinolytic response [26, 27]. This underscores that a negative D-dimer result should never delay urgent aortic imaging when clinical suspicion is high, especially in high-risk subgroups [28].

Moreover, D-dimer’s short biological half-life (<8 hours) limits its utility in subacute or chronic dissections, where levels may normalize over time. Its elevation is also nonspecific to ATAAD and can occur in disseminated intravascular coagulation (DIC), sepsis, malignancy, and postoperative states.

Given these limitations, the 2015 American College of Emergency Physicians issued a level C recommendation against using D-dimer as a standalone test for ATAAD. Integration with clinical decision tools, such as the Aortic Dissection Detection Risk Score (ADD-RS), has been shown to improve diagnostic accuracy by combining D-dimer testing with structured risk assessment. For example, the combination of ADD-RS ≤1 with a negative D-dimer result has a high negative predictive value for ruling out AAD, whereas a high ADD-RS score should prompt immediate imaging regardless of D-dimer levels [29].

Medial degeneration is the defining histological feature of ATAAD including vascular smooth muscle cell (VSMC) damage, extracellular matrix (ECM) degradation, remodeling and vascular inflammation [30]. VSMC damage leads to release of cellular proteins into circulation. Smooth muscle myosin heavy chain showed a 20-fold increase in the ATAAD patients. However, it is elevated only 3–6 hours after onset and creates a limited time window for use in the emergency department [31]. BB-isozyme of creatine kinase of the muscle, another product of VSMC injury, is elevated in the ATAAD patients but has a short time course and lacks specificity [32]. ECM supports aortic integrity, elasticity, and strength. ECM degradation and disorganization are remarkable hallmarks of ATAAD. Elastin, a major component of aortic ECM, showed elevations in the ATAAD patients. Nevertheless, only a less than 2-fold increase was observed over normal controls, making reliable clinical use questionable [33]. Matrix metalloproteinases (MMPs), particularly MMP-9, are involved in ECM degradation and are elevated in ATAAD, but their levels overlap with other vascular pathologies [34]. Although vascular inflammation is a key process in ATAAD, it may exist in other cardiovascular disorders and lacks sufficient specificity. Soluble suppression of tumorigenesis-2 (sST2), a cytokine receptor involved in the cardiovascular inflammation, showed promising diagnostic performance in a Chinese study (sensitivity 99.1%, specificity 84.9% at a 34.6 ng/mL cut-off) [35], yet these results were not replicated in European cohorts due to high inter-individual variability [36]. Other markers (such as the neutrophil-to-lymphocyte ratio) have been evaluated, yet none consistently outperform D-dimer in accuracy or reproducibility [37]. Taken together, these limitations highlight the unmet need for novel biomarkers that offer both high sensitivity and specificity, maintain stability across disease phases, and remain consistent across diverse populations.

## 3. PGs: Structure and Function

### 3.1 Basic Structure of PGs

Proteoglycans are intricate macromolecules consisting of a core protein attached by one or more GAG chains [38]. GAGs are negatively charged polysaccharides composed of repeating disaccharide units, one of which is an amino sugar (i.e., N-acetylglucosamine or N-acetylgalactosamine) and the other is a hexuronic acid (i.e., glucuronic acid or its epimer iduronic acid) [39]. The sulfation and carboxylation of these units confer a strong polyanionic character to GAGs, enabling proteoglycans to bind cations such as Na^+^ from the interstitial fluid to preserve electroneutrality. This property allows them to generate Donnan osmotic pressure, thereby retaining water and maintaining tissue hydration and compressibility [40].

Up to now, over 90 PGs subtypes have been identified with the advances in glycoproteomic techniques [41] with approximately 20 identified in the blood vessels [12]. PGs can be broadly categorized based on their GAG components, core protein characteristics and cellular locations [12] as shown in Table 1. GAG components include chondroitin sulfate (CS), dermatan sulfate (DS), keratan sulfate (KS), heparan sulfate (HS) and hyaluronan (HA) [40]. These GAGs are covalently attached to specific types of core proteins to form 4 major categories, including chondroitin sulfate proteoglycans (CSPGs) (i.e., versican and aggrecan), dermatan sulfate proteoglycans (DSPGs) (i.e., decorin and biglycan), keratan sulfate proteoglycans (KSPGs) (i.e., lumican and fibromodulin), heparan sulfate proteoglycans (HSPGs) (i.e., syndecan and perlecan) and hyaluronan-associated proteoglycans (HAPG) [39, 42]. According to cellular localization, PGs are classified into intracellular PGs (i.e., serglycin), cell-surface PGs (i.e., syndecans and glypicans) and ECM PGs. ECM PGs can further be subdivided into small leucine-rich proteoglycans (SLRPGs) such as decorin and biglycan, and hyalectans like aggrecan and versican [39]. The diversity of PGs allows them to fulfill a wide range of biological functions in various tissues and organs, including vasculatures.

**Table 1.** **The classifications of PGs**.

Classification based on GAG components, subclassification based on core proteins						
CSPG	DSPG	KSPG		HSPG		HAPG
Aggrecan (ACAN)	Decorin (DCN)	Lumican (LUM)		Syndecan	SDC1	
Versican (VCAN)	Biglycan (BGN)	Fibromodulin (FMOD)			SDC2	
Neurocan (NCAN)		Mimecan (OGN)			SDC3	
Brevican (BCAN)					SDC4	
NG2 (CSPG4)				Glypican	GPC1	
Thrombo-modulin (THBD)					GPC2	
Chondroitin sulfate proteoglycan 4 (CSPG4)					GPC3	
Inter-alpha-trypsin inhibitor heavy chain 1 (ITIH1)					GPC4	
Inter-alpha-trypsin inhibitor heavy chain 2 (ITIH2)					GPC5	
Inter-alpha-trypsin inhibitor heavy chain 5 (ITIH5)					GPC6	
Serglycin (SRGN)				Perlecan	HSPG2	
				Agrin	AGRN	
				Testican	SPOCK1	
					SPOCK2	
					SPOCK3	
					N-Tes	
Classification based on cellular localization						
Intracellular PG	Cell-surface PG		ECM PG			
Serglycin	Syndecans		SLRPG			Hyalectans
	Glypicans		Decorin (DCN)			ACAN
			Biglycan (BGN)			VCAN

PG, proteoglycan; CSPG, chondroitin sulfate proteoglycan; HSPG, heparan sulfate proteoglycan; ECM, extracellular matrix; SLRPG, small leucine-rich proteoglycan; DSPG, dermatan sulfate proteoglycan; KSPG, keratan sulfate proteoglycan; HAPG, hyaluronan-associated proteoglycans; SPOCK, SPARC/osteonectin, CWCV and Kazal-like domain proteoglycan.

### 3.2 Basic Functions of PGs in the Vasculature

PGs perform indispensable roles in maintaining ECM structure and function, contributing to vascular integrity and homeostasis. PGs play a pivotal role in organizing vascular ECM to ensure vessel elasticity and adapt to mechanical forces [38, 39]. PGs are direct space-filling molecules that regulate hydration, maintain tissue turgor through Gibbs-Donnan osmotic pressure, and provide mechanical resilience [43]. PGs may also help organize the structural framework of ECM by interacting with other ECM components. For instance, the core protein of decorin interacts with collagen fibrils to regulate their organization and diameter, enhancing tensile strength [39]. Similarly, versican interacts with hyaluronan to form hydrophilic aggregates that confer compressive resistance and are essential for vascular integrity under pulsatile flow [43].

Beyond their structural roles, PGs are central to cell signaling processes that influence vascular remodeling, inflammation, and endothelial function [12]. The negatively charged GAG chains bind to growth factors, cytokines, and chemokines, thereby modulating their local bioavailability and activity. PGs facilitate the sequestration and presentation of transforming growth factor-beta (TGF-β) and fibroblast growth factor (FGF), both of which are critical for VSMC proliferation and migration. Additionally, specific PGs regulate the bioavailability of chemokines and cytokines, influencing inflammatory responses and vascular remodeling [44]. For example, syndecans and glypicans are co-receptors for growth factors to present them in bioactive forms to recognize cognate receptors, modulating signaling pathways involved in VSMC proliferation, migration, and apoptosis [39, 45].

### 3.3 PGs in Common Vascular Diseases

The dysregulation of PGs is closely associated with vascular diseases. Alterations in the expression, composition, and distribution of PGs disrupt the ECM’s structural integrity, rendering blood vessels susceptible to mechanical stress and pathological remodeling [46, 47]. Under normal conditions, PGs constitute a minor component of the ECM, with tightly regulated interactions that maintain vessel architecture and mechanical properties. However, PGs often increase in the vascular diseases and are frequently accompanied by changes in GAG sulfation patterns [12]. In atherosclerosis, proteoglycans (i.e., versican) bind lipoproteins and promote lipid retention and the formation of atheromatous plaques [48]. PGs also modulate inflammatory responses. For example, decorin and biglycan contribute to macrophage recruitment and foam cell formation [49]. In hypertension and pulmonary arterial hypertension, PGs (i.e., glypican and decorin) worsen disease progression by interacting with endothelial cells and VSMCs to influence vascular calcification, fibrosis, and thrombosis [50, 51, 52].

## 4. PGs as Biomarkers of Medial Degeneration

Unlike intracranial or abdominal aortic aneurysms, which rarely exhibit PG accumulation, ATAAD is uniquely characterized by significant pools of PGs within regions of medial degeneration [53, 54]. These soluble fragments, reflecting the molecular disarray of the aortic wall, hold considerable promise as serum biomarkers for detecting and monitoring ATAAD. Excessive PG aggregation VSMC damage, disrupts elastic fiber organization, and fragments collagen, collectively weakening the tensile and elastic integrity of the aortic wall [55, 56, 57].

Beyond structural disruption, PG degradation products act as bioactive signaling molecules. Cleavage by MMPs and A disintegrin-like and metalloprotease domain with thrombospondin type 1 motifs (ADAMTS) family proteases yields soluble PG fragments that function as damage-associated molecular patterns (DAMPs), activating innate immune pathways and promoting macrophage and neutrophil recruitment [43, 47, 58, 59]. These immune cells further secrete proteases and reactive oxygen species, amplifying ECM injury. Non-proteolytic mechanisms, including hyaluronidase activity and macrophage-mediated release, also contribute to PG dissemination into circulation [41].

Importantly, PG synthesis itself is upregulated in response to mechanical stress and inflammatory signaling, driving maladaptive ECM remodeling [60]. Computational and biomechanical models confirm that PG-rich pools create local stress concentrations sufficient to sever elastic lamellae, impair mechanotransduction pathways critical for ECM homeostasis, and destabilize wall architecture [2, 43, 60, 61, 62]. Together, these processes leave the vessel wall structurally weakened and primed for dissection and rupture.

### 4.1 Aggrecan and Versican

Beyond their general accumulation in ATAAD, aggrecan and versican, two large aggregating CSPGs, play central roles in driving the molecular and structural changes that underlie medial degeneration [60, 63]. Both molecules possess large, highly sulfated glycosaminoglycan chains that attract water and alter tissue biomechanics by generating osmotic swelling pressures, but their impact extends beyond passive matrix disruption. Through regulated turnover, proteolytic processing, and ECM interactions, aggrecan and versican shape VSMC phenotype, inflammatory signaling, and aortic medial integrity. These multifaceted functions position them as both contributors to wall destabilization and mediators of pathways central to ATAAD pathogenesis.

#### 4.1.1 Aggrecan 

Most found in cartilaginous tissues [64], aggrecan (ACAN) is abnormally deposited in the thoracic aortic media during ATAAD [60, 63]. Its highly sulfated chondroitin and keratan sulfate GAG chains create highly hydrated microdomains that increase interlamellar spacing and compromise elastin–collagen load sharing [55, 57, 59]. Histological analyses have also demonstrated that ACAN colocalizes with elastic fiber breaks and apoptotic VSMCs [64]. Proteolytic cleavage of aggrecan by ADAMTS enzymes (e.g., ADAMTS-4, ADAMTS-5) generates neoepitopes that act as DAMPs, activating toll-like receptors (TLR2, TLR4) on immune cells and promoting secretion of interleukin-1β and tumor necrosis factor-α [65, 66]. This dual effect, mechanical Fdestabilization and inflammatory amplification, positions aggrecan as a central driver of medial degeneration.

Aberrant aggrecan accumulation has been consistently documented in thoracic aortic dissection (TAD) tissue, where it colocalizes with elastic lamellar breaks and VSMC apoptosis [60, 63, 65, 66]. Its long chondroitin and keratan sulfate chains create highly hydrated domains that expand interlamellar spacing and compromise elastin-collagen load sharing [14, 39, 47, 66]. Balanced aggrecan turnover depends on the activity of MMPs and ADAMTS proteases, specifically ADAMTS-5 activity [46, 66]. Mice lacking ADAMTS-5 exhibit impaired aggrecan cleavage, abnormal proteolytic fragment profiles, and ascending aortic anomalies as a result of ACAN accumulation [46, 65, 67]. In human TAD, upregulation of ADAMTS-1 and ADAMTS-4 further underscores dysregulated proteoglycanase activity [68]. Beyond mechanics, aggrecan peptide produced as a result of MMP and ADAMTS cleavage sites act as DAMPs, engaging toll-like receptors on immune cells to stimulate pro-inflammatory cytokine release [69, 70, 71].

While much of this work has been conducted in the context of arthritic pain and cartilage degeneration, the underlying signaling mechanisms remain relevant to vascular pathology. In particular, activation of TLR4 has been shown to promote a pro-inflammatory VSMC phenotype and to drive cellular proliferation during vascular remodeling and atherogenesis [72, 73]. Taken together, these findings support a mechanistic link by which aggrecan-derived DAMPs may contribute to VSMC dysfunction and the progression of medial degeneration in ATAAD.

#### 4.1.2 Versican

VCAN, a large chondroitin sulfate PG highly expressed in vascular tissue, also plays a prominent role in medial degeneration through its effects on ECM organization, VSMC behavior, and inflammation [12, 39, 60, 74]. In ATAAD, VCAN is enriched in areas of VSMC dropout, where it disrupts pericellular adhesion, alters fibronectin and type I collagen organization, and weakens medial tensile strength [61, 74, 75]. Its binding to hyaluronan produces bulky pericellular matrices that interfere with integrin signaling, promote VSMC detachment, and impair nutrient diffusion [74, 76, 77]. Proteolysis by ADAMTS enzymes generates versikine, a bioactive fragment that promotes apoptosis, leukocyte adhesion, cytokine release, and MMP expression [74, 78, 79, 80].

Versican accumulation is also mechanistically linked to phenotypic modulation of VSMCs. VSMC phenotypic switching is a central feature of medial degeneration, characterized by the downregulation of contractile proteins such as SM22α, alpha-smooth muscle actin (α-SMA), and myosin heavy chain 11 (MYH11), as well as the upregulation of synthetic mediators such as MMPs, osteopontin, and IL-6 [81, 82, 83]. Versican-rich pericellular matrices promote this switch by activating phosphoinositide-3-kinase–AKT (PI3K–AKT) and growth factor pathways including insulin-like growth factor (IGF) and platelet-derived growth factor (PDGF) [61, 84, 85]. In parallel, TGF-β/Sma and Mad–related proteins (SMAD) signaling modulates proteoglycan metabolism: a functional SMAD4 variant enhances PG degradation and VSMC apoptosis, thereby increasing dissection susceptibility [86]. Crosstalk between PI3K–AKT and TGF-β/SMAD pathways further amplifies VSMC phenotypic modulation, linking intracellular signaling directly to proteoglycan accumulation and ECM disarray seen in ATAAD [61, 84].

Aggrecan and versican act as both structural disruptors and signaling hubs. Their excessive accumulation imposes mechanical stress and lamellar delamination, while their proteolytic fragments amplify inflammation and promote VSMC dysfunction. By fostering phenotypic switching and impairing ECM homeostasis, these proteoglycans establish a self-reinforcing cycle of degeneration that predisposes the thoracic aorta to dissection and rupture [60, 81]. Their restricted association with thoracic aortic medial degeneration also underpins their translational potential as biomarkers: circulating aggrecan and versican fragments are detectable in acute dissection and may provide diagnostic specificity relative to other acute aortic syndromes [60, 63, 65].

### 4.2 Perlecan and Syndecan-1 (SDC-1)

HSPGs are multifunctional components of the vascular ECM that play integral roles in structural integrity, cell signaling, and mechanical homeostasis [39, 87]. Comprising a protein core modified with HS GAG chains, HSPGs are positioned to respond to both biomechanical and biochemical stimuli in the vessel wall. Among them, perlecan and SDC-1 have emerged as particularly relevant to the medial degeneration of ATAAD. These PGs not only influence the mechanical properties of the aorta but may also serve as candidate biomarkers for disease risk or activity [88, 89].

Perlecan, encoded by HSPG2, is a large HSPG broadly expressed across vascular and avascular tissues, where it localizes predominantly to basement membranes and the apical surface of cells [39]. Within the thoracic aortic wall, it is enriched at the interface of elastin and fibrillin-1 fibers in the medial layer, supporting elastic lamellae organization and matrix integrity [90]. It contributes to elastic lamellae organization, supports endothelial nitric oxide signaling [91], and maintains the contractile phenotype of VSMC [92]. The significance of perlecan in ATAAD was demonstrated in a vascular-specific perlecan knockout mouse model, in which spontaneous thoracic aortic dissection occurred with notable frequency, approximately 15% at 10 weeks and up to 38.9% by 50 weeks of age [88]. On the cellular level, perlecan deficiency was associated with significant downregulation of VSMC contractile markers (*Acta2*, *MYH11*), with preserved expression of *Myocd*, suggesting a phenotypic drift away from the contractile state. Notably, MMP activity (MMP-2 and MMP-9) was not elevated, indicating that ECM degradation was not enzymatically driven but structurally preconditioned. Given its role in maintaining elastic fiber maturity and VSMC phenotype, perlecan represents a mechanistically grounded risk determinant for medial degeneration and dissection.

However, its largely tissue-restricted localization limits its feasibility as a circulating biomarker. Instead, perlecan shows greater promise as a tissue-level marker of structural integrity or as a candidate genetic modifier in syndromic or familial aortopathies [93]. In Marfan syndrome mouse models, lower HSPG2 expression is associated with more severe cardiovascular and skeletal manifestations, indicating that variation in HSPG2 can influence disease severity [93]. In the context of syndromic or familial aortopathies, immunohistochemical or transcript-based assessment of perlecan in aortic tissue could provide insights into structural vulnerability before dissection occurs. Moreover, rare variants in HSPG2 have been reported in human connective tissue and skeletal disorders, and expression levels of HSPG2 correlate with severity in animal models. These observations indicate that HSPG2 acts not only as a structural ECM component but also as a genetic modifier influencing susceptibility to medial degeneration and dissection. Assessing perlecan in aortic tissue may therefore provide a means of preclinical ATAAD risk evaluation, supporting more precise patient stratification and informing decisions on the optimal timing of prophylactic surgery.

SDC-1 is another transmembrane HSPG expressed by VSMCs and endothelial cells, involved in modulating inflammation [94, 95, 96], ECM-cell interactions, and extracellular remodeling [94, 97, 98]. Although most of the existing literature focuses on its role in aneurysmal thoracic aorta, its regulation in ATAAD-prone tissues and relevance to dissection biology is increasingly appreciated [89]. Syndecan-1 expression was significantly elevated in the aneurysmal thoracic aorta: 3.6-fold at the mRNA level and 2.3-fold at the protein level relative to healthy aortas. This pattern of upregulation suggests that SDC-1 is a component of the medial remodeling response, potentially reflecting VSMC activation under hemodynamic stress. However, mechanistic interrogation in a murine model designed to induce dissection through β-aminopropionitrile (BAPN)-mediated ECM weakening and angiotensin II (Ang II) infusion revealed that SDC-1 is not required for the development or severity of thoracic aortic dissection [89]. Mice deficient in syndecan-1 exhibited comparable rates of ATAAD incidence, rupture, and survival compared to wild-type controls after both short-term and chronic Ang II exposure. Furthermore, no differences were observed in elastin degradation, collagen deposition, or immune cell infiltration. These findings suggest that SDC-1 may function more as a reactive marker of medial stress rather than a causal mediator of dissection. Nonetheless, its reproducible upregulation in dissection-prone tissue raises the possibility of diagnostic utility. If shown to be reliably elevated in plasma during acute or subacute dissection, SDC-1 could complement existing biomarkers by reflecting cellular and ECM-level stress responses. Future studies should clarify whether circulating SDC-1 levels can reliably distinguish dissection from other acute cardiovascular syndromes and define their temporal expression profile.

### 4.3 Biglycan and Decorin

Biglycan and decorin, archetypal class I SLRPs, share conserved structural domains including leucine-rich repeats and chondroitin/dermatan sulfate glycosaminoglycan side chains, allowing them to bind collagen, modulate TGF-β activity, and regulate matrix assembly [99]. These PGs are widely expressed across connective, vascular, and immune tissues, serving critical roles in matrix stabilization and cellular signaling. Biglycan is distributed throughout all layers of the aortic wall, while decorin is primarily localized to the adventitia. Both facilitate collagen fibrillogenesis and participate in the biomechanical maintenance of the vessel wall. Their physiological relevance is underscored in human population and knockout animal models. In humans, loss-of-function mutations in the *BGN* gene encoding biglycan have been associated with syndromic forms of ATAAD, frequently presenting with early-onset dissection, skeletal abnormalities, and elevated TGF-β signaling in affected aortic tissues [100]. Given that both biglycan and decorin are capable of binding and sequestering TGF-β, their loss is thought to facilitate medial degeneration via dysregulated growth factor activity and matrix degradation. Supporting this, reduced decorin transcript levels have been reported in medial samples from patients with sporadic, non-syndromic thoracic aortic aneurysms, suggesting an acquired or context-specific role in non-genetic disease as well. In murine model, mice lacking either biglycan or decorin exhibit reduced collagen fibril diameter and disorganized ECM architecture, with biglycan-deficient males experiencing spontaneous thoracic aortic rupture due to profound structural compromise [100, 101].

These mechanistic roles, coupled with evidence of their proteolytic release during vascular injury, raise the possibility that biglycan and decorin may serve as measurable serum biomarkers of acute aortic pathology. Soluble biglycan has been identified as a DAMP, capable of activating TLR2 and TLR4 signaling and promoting sterile inflammation [102, 103, 104], a hallmark of acute aortic syndromes. Elevated circulating levels of biglycan have been documented in a range of inflammatory and cardiovascular conditions, including atherosclerosis [105, 106], pulmonary hypertension [107] and chronic liver disease [108], suggesting its potential as a nonspecific indicator of vascular ECM remodeling. Decorin may behave similarly, although data on its serum dynamics in aortic pathology are still limited.

However, several challenges currently impede the clinical translation of these molecules as biomarkers. Neither biglycan nor decorin is specific to the aorta or to dissection, and both are altered in a variety of systemic inflammatory and fibrotic states. No human studies have yet investigated the utility of biglycan or decorin as serum biomarkers for ATAAD. Most existing findings derive from tissue analyses or animal models, and the lack of standardized serum assays further limits clinical translation. Consequently, while these molecules have compelling mechanistic links to medial degeneration, their value as circulating biomarkers for acute aortic dissection remains entirely theoretical, highlighting a clear gap in human-focused research.

## 5. PGs as Serum Biomarkers in ATAAD

### 5.1 Aggrecan and Versican

Among the promising candidates, aggrecan and versican, two large aggregating CSPGs, have demonstrated significant potential [60, 63, 109]. Their diagnostic value arises from their physiological roles in maintaining ECM integrity and their pathological accumulation and release during aortic wall remodeling and degeneration. In the aorta, aggrecan and versican localize to the tunica media, contributing to its mechanical properties and ability to withstand hemodynamic stress [39]. Aggrecan and versican contribute distinct yet complementary roles to vascular biology. Aggrecan, traditionally associated with cartilage, maintains tissue hydration, and resists compressive forces through its extensive GAG chains [39]. Although its expression in the vasculature was initially underappreciated, proteomic studies have confirmed its presence in the aorta, where it contributes to ECM stability [110]. Versican, ubiquitously expressed across tissues such as the adult brain and large vessels, regulates VSMC adhesion, proliferation, and migration [39, 111].

The elevated levels of aggrecan and versican have been shown to distinguish ATAAD from other conditions. In a pivotal study by König *et al*. [63], serum plasma levels of aggrecan were systematically evaluated in ATAAD through a two-phase design, first using a pilot cohort and then validating the findings in an expanded population. The results of the initial phase revealed a significant elevation in plasma aggrecan levels in ATAAD patients, with concentrations four to five times higher than those in both control groups. Specifically, the mean plasma aggrecan level in ATAAD patients (n = 14) was 50.16 ± 5.43 ng/mL, compared to 10.33 ± 1.42 ng/mL in healthy controls and 11.92 ± 1.77 ng/mL in patients who underwent minimally invasive mitral valve repair. An expanded cohort that enrolled 33 ATAAD patients confirmed these findings, showing nearly a ten-fold increase in aggrecan levels in ATAAD patients (38.59 ± 4.08 ng/mL) compared to those with chronic asymptomatic aneurysms (4.45 ± 0.90 ng/mL). Furthermore, aggrecan levels in ATAAD patients were significantly higher than in STEMI patients (11.77 ± 1.89 ng/mL) and those without coronary artery disease (8.88 ± 1.8 ng/mL). These findings collectively provide compelling evidence that plasma aggrecan levels are markedly elevated in ATAAD patients and are distinct from levels observed in other differential diagnoses, such as myocardial infarction and chronic aneurysms.

Aggrecan demonstrates high diagnostic specificity for ATAAD, with serum levels unaffected by age, sex, aortic root involvement, or dissection extent. Its levels also remain unchanged in non-dissecting ascending aortic aneurysms, excluding its utility as a screening marker for chronic aneurysmal disease. The significance of these findings is further amplified by the results of receiver operating characteristic (ROC) curve analysis, which determined an optimal diagnostic threshold of 14.3 ng/mL. At this level, aggrecan demonstrated a sensitivity of 97% and a specificity of 81%, far exceeding the specificity of widely used biomarkers such as D-dimer (53%) [112]. The high specificity is essential in the emergency, where misdiagnosis may result in inappropriate treatments that exacerbate patient outcomes. Moreover, aggrecan’s diagnostic utility is enhanced by its stability. Aggrecan levels remain elevated for up to 72 hours post-symptom onset, providing a wide diagnostic window even in delayed presentations—a common challenge in ATAAD diagnosis​ [113].

Although aggrecan demonstrates better performance than markers D-dimer, its specificity and sensitivity remain inferior to high-sensitive troponin assays used in myocardial infarction. [Additionally, the diagnostic accuracy reported by König *et al*. [63] is based on a relatively small cohort of 33 non-consecutive, hemodynamically stable ATAAD patients, all with Stanford type A dissections. While the cohort included both sexes in nearly equal proportions and reflected common cardiovascular risk factors such as hypertension, hyperlipidemia, diabetes, obesity, and nicotine use, these features limit the generalizability of the findings to broader, more diverse patient populations, including those with type B dissections or differing comorbidity profiles.] As a major component of cartilage, aggrecan is also prone to degradation in osteoarthritis [69], raising concerns about diagnostic confounding, especially in broader clinical populations.

The diagnostic value of versican is revealed by proteomic studies using liquid chromatography–tandem mass spectrometry (LC-MS/MS) Elevation of versican and its proteolytic fragments, such as the DPEAAE neoepitope, a versican-specific cleavage product, was consistently detected in ATAAD aortic tissue and blood circulation [60]. Immunohistochemical analyses revealed intense versican staining across multiple lamellar units in the tunica media, with the highest concentrations in regions of medial degeneration. The release of versican fragments into the bloodstream during ECM remodeling provides an additional layer of diagnostic utility, potentially enabling real-time monitoring of disease activity and progression​.

Unlike aggrecan, versican is also elevated in non-dissected aneurysmal thoracic aorta. In a transcriptomic study conducted by our team, versican-positive myofibroblasts were identified as critical intermediates in the pathological transition of VSMC from a contractile to a synthetic phenotype [61]. Besides, versican presents similar challenges due to its involvement in various pathological conditions, further limiting disease specificity. For example, ATAAD may be secondary to thoracic aortic dilation and aneurysm. The elevation of versican in aneurysmal thoracic aorta limits its utility in distinguishing ATAAD and thoracic aortic aneurysms (TAAs). Future efforts to improve specificity could focus on characterizing the unique versican degradome in ATAAD. Quantifying specific neoepitopes, such as those generated by ADAMTS proteases versus other proteinases (e.g., MMPs), may reveal a fragment signature unique to acute dissection, providing an actionable strategy for biomarker development.

### 5.2 Lumican

SLRPs are a family of ECM molecules that play diverse roles in tissue homeostasis, from collagen fibrillogenesis to the regulation of inflammation and signal transduction [114, 115, 116]. Among these molecules, lumican (LUM), a class II SLRP, has garnered attention for its involvement in the vascular remodeling and aortic wall homeostasis, particularly in the context of ATAAD [39]. LUM plays a role in the ECM architecture by controlling collagen fibril assembly and by interacting with other matrix constituents to uphold vascular stability [117, 118, 119]. In the context of ATAAD, heightened proteolytic activity cleaves ECM components, releasing LUM into the circulation and thereby linking structural aortic degeneration to elevated serum levels of this molecule [120, 121, 122, 123]. Such dysregulation of LUM demonstrates its potential utility as a biomarker for early diagnosis, stratification of disease severity, and prognostic evaluation in ATAAD.

Initial proteomic investigations into serum alterations in ATAAD first highlighted LUM’s diagnostic potential [122, 123]. Using isobaric tags for relative and absolute quantification (iTRAQ), researchers identified LUM as a significantly elevated ECM protein in ATAAD patients compared to both healthy controls and AMI patients. Serum concentrations of LUM in ATAAD patients (2.66 ± 4.58 ng/mL) were markedly higher than in controls (0.85 ± 0.53 ng/mL) and acute myocardial infarction (AMI) patients (0.69 ± 0.34 ng/mL) [121]. These findings suggested that LUM could be a specific marker for aortic pathology, differentiating it from markers associated with cardiac injury or general vascular dysfunction. Efforts to refine LUM’s diagnostic performance progressed with the integration of complementary proteomic approaches. In 2022, a study combining iTRAQ and label-free techniques identified LUM as consistently elevated in ATAAD patients (n = 77) relative to controls (n = 73), with a standalone area under the receiver operating characteristic curve (AUC) of 0.636 [122].

While moderate in isolation, LUM’s diagnostic accuracy improved significantly when combined with complementary biomarkers, such as peptidase inhibitor 16 (PI16) and fibrinogen-like protein 1 (FGL1) [122]. The selection of PI16 and FGL1 to complement Lumican in a multimarker panel was driven by a targeted proteomic discovery process. These candidates were identified through a systematic screening approach using iTRAQ and label-free proteomics on serum from ATAAD patients, which revealed both PI16 and FGL1 as significantly upregulated proteins [122]. Their pathobiological interactions are distinct yet synergistic in the context of ATAAD pathogenesis. PI16, a peptidase inhibitor, is postulated to modulate inflammatory and proteolytic processes that contribute to the degradation of the aortic wall’s structural integrity by inhibiting MMPs and consequently driving the accumulation of PGs [124, 125]. Conversely, FGL1 is implicated in dysregulated hemostasis and enhances TGF-β1-mediated fibrotic signaling, a pathway critically involved in aortic wall injury and repair [122]. This combination contributes to the panel’s improved diagnostic performance by simultaneously interrogating the multifactorial pathology of ATAAD: Lumican reflects extracellular matrix remodeling, PI16 adds a dimension of inflammatory proteolysis, and FGL1 captures associated coagulation and fibrotic activation.

The validity of this combinatorial approach is supported by the panel’s significantly enhanced AUC of 0.780, with sensitivity and specificity values of 67.5% and 76.7%, respectively [122]. This demonstrates superior discriminatory power compared to any single marker alone, as the integration of biomarkers from complementary pathways yields greater diagnostic specificity and a more robust reflection of the disease’s complex biology. This successful combination establishes a blueprint for integrating other emerging biomarkers (e.g., ACAN, sST2, etc.) into even more comprehensive panels. This could lead to the development of a highly accurate, rapid biomarker assay that not only aids in diagnosis but also helps stratify rupture risk and guide personalized therapeutic strategies, fundamentally improving ATAAD management.

Beyond initial diagnostic associations, subsequent research explored how circulating LUM levels may relate to clinical severity in ATAAD, correlating serum LUM levels with vascular involvement using multi-slice computed tomography angiography (MSCTA) [120]. Elevated LUM levels were observed in ATAAD patients (2.32 ± 4.29 ng/mL) compared to controls (0.85 ± 0.53 ng/mL) and patients with intramural hematoma (IMH) (0.72 ± 0.32 ng/mL). Notably, higher LUM levels were correlated with greater disruption of the aortic media and more extensive branch-vessel involvement. In addition to differentiating ATAAD cases from controls, elevated LUM concentrations reflect disease severity and anatomical complexity [120]. These findings suggest that LUM can serve as a surrogate biomarker in scenarios where advanced imaging is inaccessible or delayed, and support its prospective role in longitudinal monitoring of patients with chronic aortic pathology at risk for acute dissection.

As research progressed, attention also turned to LUM’s potential role in predicting clinical outcomes and disease prognosis. In a 2021 study of 58 patients undergoing surgical repair for ATAAD, Hsu *et al*. [126] demonstrated that elevated preoperative serum LUM concentrations, quantified via enzyme-linked immunosorbent assay (ELISA), were significantly associated with an increased risk of prolonged mechanical ventilation (>72 hours) and extended hospitalization (>30 days). A threshold of 1.547 ng/mL identified patients at risk for prolonged ventilation with 100% sensitivity and 40.4% specificity, while a threshold of 5.992 ng/mL predicted extended hospitalization with 58% sensitivity and 91.3% specificity. These findings position LUM as a potential biomarker of disease severity and postoperative risk, with clinical utility in informing perioperative risk stratification and guiding the targeted allocation of intensive care resources. Beyond the perioperative period, evidence from Harrison *et al*. [127] suggests that serum LUM levels may also reflect long-term aortic pathology, as patients with bicuspid aortic valves who later developed aneurysms exhibited low variance in LUM concentrations, while those without aneurysms showed high variance. Although the precise pathophysiological mechanisms remain unclear and validation in larger cohorts is needed, these observations imply that LUM could have predictive value for long-term outcomes such as late aortic events or re-dissection.

Collectively, these findings highlight the complementary diagnostic potential of different PGs in ATAAD. A comparative summary of their vascular roles, serum alterations, and diagnostic performance is provided in Table 2 (Ref. [44, 60, 61, 63, 64, 66, 80, 88, 89, 91, 92, 94, 95, 96, 99, 101, 102, 108, 111, 114, 116, 120, 122]).

**Table 2.** **Comparative summary of PGs in ATAAD**.

Proteoglycan	Type/Family	Key roles in vasculature	Serum elevation in ATAAD	Diagnostic performance	Specificity for ATAAD	Limitations	References
Aggrecan	CSPG/Hyalectan	ECM hydration, load resistance, structural integrity	Markedly elevated (up to 10× in ATAAD)	Sensitivity: 97% Specificity: 81% (cutoff: 14.3 ng/mL)	High; low in STEMI and aneurysm	Confounding from cartilage diseases (e.g., osteoarthritis); not useful for screening chronic aneurysms	[60, 63, 64, 66]
Versican	CSPG/Hyalectan	VSMC migration, ECM remodeling, signaling	Elevated in ATAAD and aneurysm tissues	No standardized serum cutoff; detected in LC-MS/MS and IHC studies	Moderate; expression is also high in cancer/fibrosis	Lacks ATAAD-specific signature; needs neoepitope or fragment-level profiling	[60, 61, 80, 111]
Lumican	SLRP (Class II)	Collagen fibrillogenesis, ECM integrity	Elevated in ATAAD vs. controls and AMI	AUC alone: 0.636 AUC (3-marker panel): 0.780	Moderate; reflects ECM remodeling	Limited sensitivity when used alone; better in multimarker panels	[114, 116, 120, 122]
Perlecan	HSPG (Basement Membrane)	Elastic lamellae organization, VSMC phenotype maintenance	No direct serum data; increased risk in KO mice	Not applicable	Low; best studied at tissue/genetic level	Not suitable as a serum biomarker; may be better as a structural or genetic marker	[88, 91, 92]
Syndecan-1 (SDC-1)	HSPG (Transmembrane)	Inflammatory modulation, ECM-cell interaction	Upregulated in aneurysmal thoracic tissue; serum data lacking	No validated serum performance data	Low; overexpressed in many vascular injuries	Currently lacks diagnostic value; may reflect stress response rather than disease cause	[89, 94, 95, 96]
Biglycan/Decorin	SLRP (Class I)	Collagen crosslinking, TGF-β sequestration	Evidence from animal/tissue studies only	Not validated in serum	Low; altered in various systemic diseases	Limited human data; no established serum assay or kinetics in ATAAD	[44, 99, 101, 102, 108]

AUC, area under the receiver operating characteristic curve; AMI, acute myocardial infarction; ATAAD, acute type A aortic dissection; CSPG, chondroitin sulfate proteoglycan; ECM, extracellular matrix; HSPG, heparan sulfate proteoglycan; IHC, immunohistochemistry; KO, knockout (mice); LC-MS/MS, liquid chromatography–tandem mass spectrometry; SLRP, small leucine-rich proteoglycan; STEMI, ST-elevation myocardial infarction; TGF-β, transforming growth factor-β; VSMC, vascular smooth muscle cell.

## 6. Future Perspectives

PGs are emerging as promising serum biomarkers for ATAAD, reflecting underlying ECM disruption. Elevated levels of aggrecan, versican, and lumican in affected patients suggest these molecules may offer diagnostic value beyond traditional markers such as D-dimer. Their greater specificity could facilitate more accurate differentiation of ATAAD from other acute cardiovascular syndromes, particularly when incorporated into tiered diagnostic algorithms or used in conjunction with high-sensitivity assays.

ATAAD may derive from both normal and aneurysmal thoracic aorta with different ages, genders and severity, which emphasizes the heterogeneity of ATAAD. For example, chronic aortic dilation and aneurysm lead to progressive ECM remodeling and PGs accumulation. Future research should aim to clarify the pathophysiological role of PGs in ATAAD by exploring their differential expression across previous aortic diameters, age groups, disease severity, and dissection stages. Characterizing these dynamics may improve risk stratification and inform stage-specific biomarker development.

In parallel, investigating PG metabolites particularly those produced by ADAMTS-mediated cleavage could uncover novel diagnostic or therapeutic targets [46]. In parallel, investigating PG metabolites, particularly those produced by ADAMTS-mediated cleavage, could reveal novel diagnostic or therapeutic targets. Key ADAMTS isoforms implicated in PG degradation in ATAAD include ADAMTS-1, ADAMTS-4, and ADAMTS-5 [41, 46, 67, 68]. ADAMTS-1 and ADAMTS-4 cleave versican, contributing to ECM remodeling, while ADAMTS-5 (aggrecanase-2) targets aggrecan and is elevated in thoracic aortic aneurysm and dissection tissues. Understanding the roles of these isoforms could guide future studies on PG-based biomarkers and potential therapeutic interventions.

Correlating tissue expression with serum levels and clinical outcomes will be essential for validating PG utility as circulating biomarkers. Integrating PGs with established markers like D-dimer may further enhance diagnostic precision, but this approach requires confirmation through large, multicenter studies before translation into clinical practice. Harnessing the diagnostic and prognostic power of PGs could mark a turning point in ATAAD care, transforming current paradigms through precision-driven, evidence-based strategies.

## 7. Conclusion

PGs are emerging as biologically meaningful serum biomarkers for TAD, directly reflecting ECM disruption rather than systemic fibrinolysis alone. Their release into circulation during medial degeneration provides a mechanistic link between molecular pathology and measurable serum signals, distinguishing them from established but nonspecific markers such as D-dimer.

Among the leading candidates, aggrecan and versican capture osmotic and structural perturbations within the aortic wall, while lumican reflects collagen remodeling and disease severity. Collectively, these molecules demonstrate superior specificity compared to conventional biomarkers and offer the potential to differentiate TAD from acute coronary syndromes or pulmonary embolism, conditions that frequently confound diagnosis. Emerging evidence also suggests prognostic applications, with circulating proteoglycan levels correlating with disease extent and postoperative outcomes.

Nevertheless, significant challenges remain before clinical translation can be achieved. The pathways governing proteoglycan release and clearance are incompletely understood, and current assays lack standardization across platforms. Large, prospective, multicenter studies are needed to define diagnostic thresholds, validate reproducibility, and evaluate performance across diverse populations. Furthermore, their integration into multimarker panels alongside D-dimer may provide the greatest diagnostic yield, balancing sensitivity with enhanced specificity.

In sum, proteoglycans hold promise to advance precision diagnostics in TAD, bridging molecular pathology with clinical practice. Their successful validation could transform current diagnostic frameworks, enabling earlier detection, improved risk stratification, and targeted perioperative management, ultimately improving patient survival and long-term outcomes.
